# Low Birth Weight Is a Risk Factor for Severe Retinopathy of Prematurity Depending on Gestational Age

**DOI:** 10.1371/journal.pone.0109460

**Published:** 2014-10-15

**Authors:** Pia Lundgren, Anna Kistner, Eva M. Andersson, Ingrid Hansen Pupp, Gerd Holmström, David Ley, Aimon Niklasson, Lois E. H. Smith, Carolyn Wu, Ann Hellström, Chatarina Löfqvist

**Affiliations:** 1 Institute of Neuroscience and Physiology, Sahlgrenska Academy at University of Gothenburg, Gothenburg, Sweden; 2 Institution of Molecular Medicine and Surgery, Karolinska Institutet, Stockholm, Sweden; 3 Department of Occupational and Environmental Medicine, Sahlgrenska Academy at University of Gothenburg, Gothenburg, Sweden; 4 Department of Pediatrics, Institute of Clinical Sciences, Lund University and Skane University Hospital, Lund, Sweden; 5 Department of Neuroscience, Ophthalmology, Uppsala University, Uppsala, Sweden; 6 Department of Pediatrics, Institute of Clinical Sciences, Sahlgrenska Academy at University of Gothenburg, Gothenburg, Sweden; 7 Department of Ophthalmology, Boston Children's Hospital, Harvard Medical School, Boston, Massachusetts, United States of America; Massachusetts Eye & Ear Infirmary, Harvard Medical School, United States of America

## Abstract

**Objective:**

To evaluate the impact of low birth weight as a risk factor for retinopathy of prematurity (ROP) that will require treatment in correlation with gestational age at birth (GA).

**Study design:**

In total, 2941 infants born <32 weeks GA were eligible from five cohorts of preterm infants previously collected for analysis in WINROP (Weight IGF-I Neonatal ROP) from the following locations: Sweden (EXPRESS) (*n* = 426), North America (*n* = 1772), Boston (*n* = 338), Lund (*n* = 52), and Gothenburg (*n* = 353). Data regarding GA at birth, birth weight (BW), gender, and need for ROP treatment were retrieved. Birth weight standard deviation scores (BWSDS) were calculated with Swedish as well as Canadian reference models. Small for gestational age (SGA) was defined as BWSDS less than −2.0 SDS using the Swedish reference and as BW below the 10^th^ percentile using the Canadian reference charts.

**Results:**

Univariate analysis showed that low GA (*p*<0.001), low BW (*p*<0.001), male gender (*p*<0.05), low BWSDS_Canada_ (*p*<0.001), and SGA_Canada_ (*p*<0.01) were risk factors for ROP that will require treatment. In multivariable logistic regression analysis, low GA (*p*<0.0001), male gender (*p*<0.01 and *p*<0.05), and an interaction term of BWSDS*GA group (*p*<0.001), regardless of reference chart, were risk factors. Low BWSDS was less important as a risk factor in infants born at GA <26 weeks compared with infants born at GA ≥26 weeks calculated with both reference charts (BWSDS_Sweden_, OR = 0.80 vs 0.56; and BWSDS_Canada_, OR = 0.72 vs 0.41).

**Conclusions:**

Low BWSDS as a risk factor for vision-threatening ROP is dependent on the infant's degree of immaturity. In more mature infants (GA ≥26 weeks), low BWSDS becomes a major risk factor for developing ROP that will require treatment. These results persist even when calculating BW deficit with different well-established approaches.

## Introduction

Retinopathy of prematurity (ROP) is a disease affecting very preterm infants that can potentially result in blindness. Timely detection and treatment of ROP is crucial. It is important to determine all risk factors in order to improve the identification of infants at greatest risk for severe ROP.

The most important risk factors for ROP are the degree of prematurity [1] and low birth weight (BW) [2], but there are other risk factors associated with infant postnatal morbidity such as days of ventilation [3], sepsis [4], hyperglycemia [5], blood transfusions [6], and bronchopulmonary dysplasia [7].

In recent years, studies have consistently identified poor postnatal weight gain as a strong predictor of ROP [8]–[12]. Study findings have, however, been contradictory as to whether or not prenatal growth restriction is a risk factor for ROP. Prenatal growth restriction can be defined as the infant's deficit from normal birth weight standard deviation score (BWSDS). The term small for gestational age (SGA), defined as BW per GA below a certain percentile or confidence interval based on growth charts, is also frequently used to describe infants' prenatal growth restriction. SGA was found to be a risk factor for ROP in some studies [2], [13]–[15]. However, in other studies no significant differences were found between infants born SGA and those with a BW appropriate for their gestational age and the risk of developing ROP [3], [16]–[18]. A possible explanation for these inconsistent results may be differences in the characteristics of the study populations and study designs. The definition of SGA has varied in previous studies where it has been defined as a BW ranging from below the 3^rd^ (approximately corresponding to 2 SD below the gestational-age related mean) to below the 10^th^ percentile. Furthermore, the definition of normal BW in relation to GA varies according to different growth charts. Growth charts used throughout the world vary in design; some are based on longitudinal fetal ultrasound weight estimations and thereby aim to reflect undisturbed intrauterine growth [19], [20], some are based on live births [21], [22], and others on live as well as still births [23].

Therefore, the aim of this study was to clarify the association between low BW and the development of ROP that will require treatment in a large cohort of very preterm infants. Calculation of BW deficit and definition of SGA was performed according to different reference models of GA-related growth to determine if this would affect the results.

Our major findings were that low BWSDS is a risk factor for preterm infants who will require treatment for ROP and that the impact of low BWSDS in more mature infants who will require ROP treatment is greater. The results persisted independent of differences in the growth charts applied.

## Study Population and Methods

### Study population

For this study, data from five cohorts enrolled in the WINROP (Weight IGF-1 Neonatal ROP) studies were retrospectively reviewed. These were: the *EXPRESS cohort*, extremely preterm (GA <27 weeks, *n* = 707) infants born in Sweden between 2004 and 2007 [24]; *North American multicenter cohort*, preterm infants (*n* = 1965) born at ten level III neonatal intensive care units in the USA and Canada between 2006 and 2009 [25]; *Boston cohort*, preterm infants (*n* = 374) born at Brigham and Women's Hospital, Boston (USA) between 2005 and 2008 [26]; *Lund cohort*, preterm infants (*n* = 60) born at Skane University Hospital, Lund (Sweden) between 2005 and 2007 [11]; and *Gothenburg cohort*, preterm infants (*n* = 354) screened and/or treated for ROP at Sahlgrenska University Hospital, Gothenburg (Sweden) between 2004 and 2007 [10]. Comprehensive descriptions of all infants and data collected in each cohort have been reported previously [10], [11], [24]–[26].

**Figure 1:**
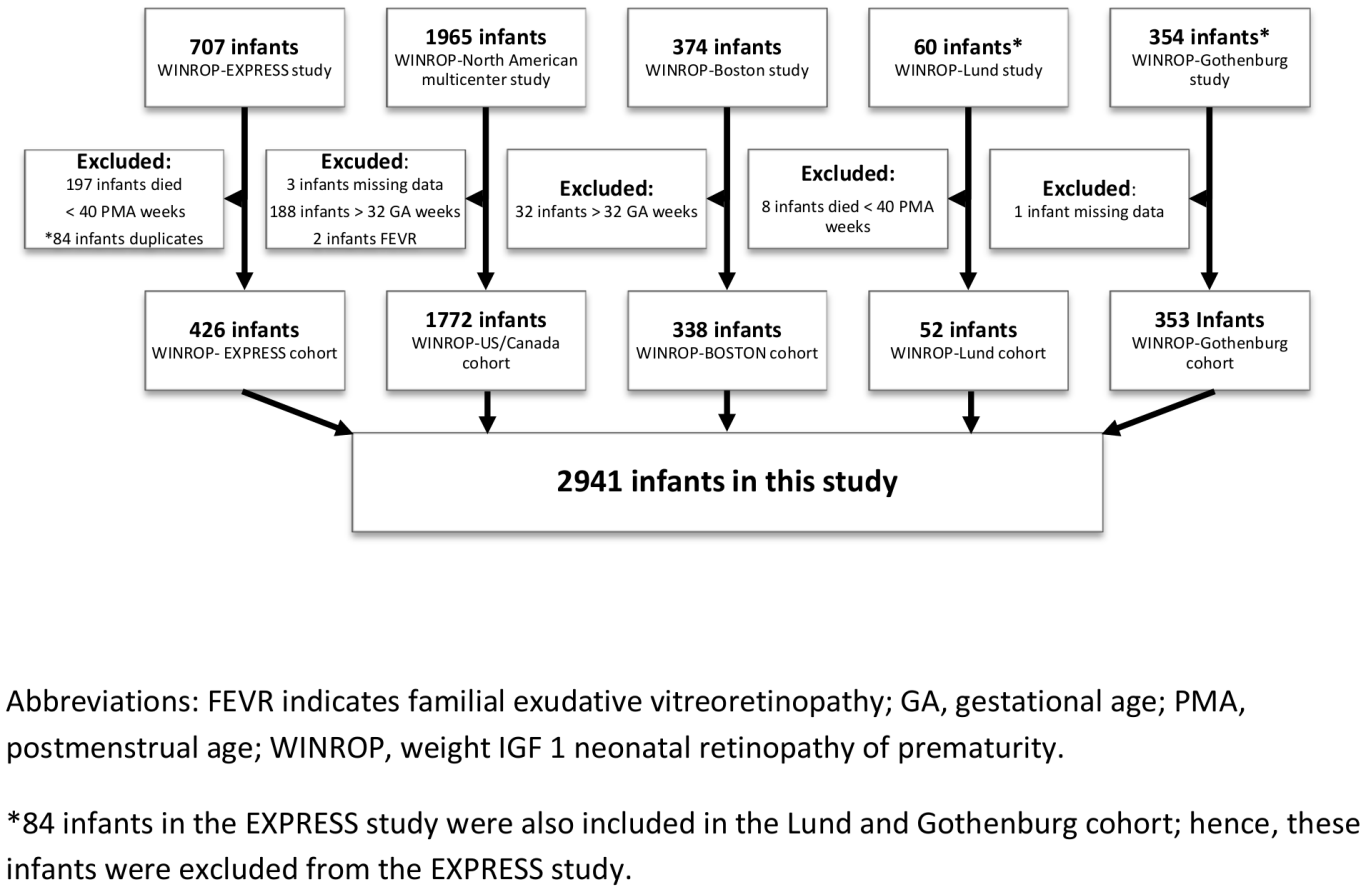
Flowchart of the study population.

Data regarding correlations between ROP requiring treatment and GA, BW, and gender were retrieved retrospectively from each original study. In the current analysis, infants were excluded if they were born after 32 week GA. Infants who died before 40 weeks postmenstrual age were also excluded since ROP grading may have been incomplete. Due to different inclusion criteria in the cohorts, the number of infants excluded due to death before 40 weeks postmenstrual age differed. The number of infants excluded in each cohort is presented in Figure 1.

### ROP screening and treatment

ROP screening examinations were conducted according to current national guidelines for each cohort. The revisited International Classification of Retinopathy of Prematurity was used for ROP classification in all cohorts [27]. Details on current ROP screening guidelines and schedules and the performance of the ROP examinations are described in each previous publication [1], [10], [11], [24]–[26]. The recommendations of the Early Treatment for Retinopathy of Prematurity Cooperative Group were followed for treatment [28].

### Statistical analysis

Birth weight standard deviation scores (BWSDS) were calculated using two well-established growth charts, both developed for infants born after 22 weeks GA. The Swedish gender-specific reference is considered to reflect undisturbed intrauterine growth [19], and the Canadian gender-specific reference is based on live as well as still births [23]. SGA was defined as BW less than −2.0 BWSDS based on the Swedish growth reference and as BW below the 10^th^ percentile based on the Canadian growth reference. We further defined severe growth restriction as a BW below the 3^rd^ percentile using the Canadian growth reference.

The outcome indicator variable was whether or not the infants required ROP treatment. Logistic regression was used to analyze risk factors. Infants were divided into five GA week groups; infants born at GAs of 22–23 weeks, 24–25 weeks, 26–27 weeks, 28–29 weeks, and 30–31 weeks. For infants born GA ≥26 weeks, all variables reflecting prenatal growth restriction, regardless growth reference or definition, were highly significant as risk factors as follows: BWSDS_Sweden_ for GA 26–27 weeks and for GA 28–29 weeks (*p-values* <0.01); SGA_Sweden_ for GA 26–27 weeks (*p*<0.001); BWSDS_Canada_ for GA 26–27 weeks and for GA 28–29 weeks (*p-values*<0.01); SGA_Canada_ for GA 26–27 weeks and for GA 28–29 weeks (*p-values*<0.01); severe growth restriction (Canada) for GA 26–27 weeks and for GA 28–29 weeks (*p*<0.001). To assess whether BWSDS had a different impact on ROP requiring treatment for infants born at GA <26 weeks compared with infants born at GA ≥26 weeks, we allowed for different associations with BWSDS by including an interaction term (BWSDS*GA group, where GA group = 1 if GA ≥26 weeks and 0 if GA <26 weeks). The multivariable logistic regression analysis included GA, gender, BWSDS, the indicator variable GA group, and the interaction term BWSDS*GA group. Two different BWSDS were calculated; one using the Swedish growth reference and one using the Canadian growth reference. Hence, two multivariable logistic regression models were estimated.

IBM SPSS Statistics 20 for Microsoft Windows (IBM Corporation, Armonk, NY) was used for statistical analysis as well as SAS version 9.3. Mann-Whitney U test was used for group comparisons. Correlations were assessed using the Spearman correlation coefficient (r_S_). Wilcoxon Signed Rank test was used to compare the two BWSDS references. The association with the outcome (ROP requiring treatment or no ROP treatment) is expressed as the odds ratios (OR) with 95% confidence intervals (CI). The Hosmer and Lemeshow test was used to assess the goodness-of-fit for the multivariable logistic regression model [29].

### Ethics statement

The Swedish studies were approved by the Regional Ethical Review Board of Gothenburg, Sweden. The American studies were retrospective chart reviews; hence no consent for data retrieval was needed.

## Results

### Study population

For this study, 2941 infants were eligible. Infants were from the EXPRESS cohort (426 infants), North American cohort (1772 infants), Boston cohort (338 infants), Lund cohort (52 infants), and the Gothenburg cohort (353 infants) (Figure 1). The birth characteristics of infants in each cohort/GA group are described in Table 1.

**Table 1:** Birth characteristics of infants enrolled in the study by cohort and GA group.

COHORT	No. infants	GA weeks+days median (range)	BW grams median (range)	Swedish reference BWSDS median (range)	SGA BWSDS <−2.0 SDS Swedish reference	Canadian reference BWSDS median (range)	SGA BW <10^th^ percentile Canadian reference	Severe growth restriction BW <3^rd^ percentile Canadian reference	Male gender % (*n*)	ROP requiring treatment % (*n*)
**EXPRESS**	426	25+4/7	780	−0.68	16.9%	0.13	9.4%	2.1%	54.0%	17.1%
		(22+1/7 to 26+6/7)	(361–1315)	(−5.18 to 3.88)	(72/426)	(−2.69 to 2.87)	(40/426)	(9/426)	(230/426)	(73/426)
**North**	1772	28+0/7	1010	−1.34	31.6%	−0.20	13.7%	3.8%	52.5%	9.8%
**American**		(22+5/7 to 31+6/7)	(378–2240)	(−6.62 to 3.35)	(560/1772)	(−3.27 to 2.87)	(242/1772)	(67/1772)	(931/1772)	(173/1772)
**Boston**	338	28+5/7	1050	−1.31	34.0%	−0.11	13.9%	4.7%	54.7%	4.1%
		(23+1/7 to 31+6/7)	(450–2400)	(−5.18 to 4.47)	(115/338)	(−2.58 to 3.07)	(47/338)	(16/338)	(185/338)	(14/338)
**Lund**	52	26+1/7	850	−0,77	25.0%	−0.01	7.7%	3.8%	51.9%	17.3%
		(23+0/7 to 30+5/7)	(448–1716)	(−5.00 to 0.89)	(13/52)	(−3.07 to 1.30)	(4/52)	(2/52)	(27/52)	(9/52)
**Gothenburg**	353	29+4/7	1290	−1.04	22.4%	0.02	11.0%	2.3%	59.2%	7.4%
		(23+3/7 to 31+6/7)	(425–2210)	(−5.59 to 2.50)	(79/353)	(−2.88 to 2.47)	(39/353)	(8/353)	(209/353)	(26/353)
**GA GROUP**										
**GA 22–23**	126	23+4/7	582	−0.46	5.6%	0.13	4.0%	0%	52.4%	45.2%
**wk**		(22+1/7 to 23+6/7)	(361–925)	(−3.27 to 3.88)	(7/126)	(−1.56 to 2.87)	(5/126)		(66/126)	(57/126)
**GA 24–25**	690	25+0/7	720	−0.84	18.0%	−0.06	10.6%	2.9%	55.2%	24.8%
**wk**		(24+0/7 to 25+6/7)	(348–1154)	(−5.00 to 2.49)	(124/690)	(−3.07 t0 2.41)	(73/690)	(20/690)	(381/690)	(171/690)
**GA 26–27**	714	26+6/7	920	−1.16	26.5%	−0.03	11.3%	3.8%	51.4%	8.1%
**wk**		(26+0/7 to 27+6/7)	(398–1430)	(−5.43 to 2.27)	(189/714)	(−2.87 to 2.28)	(81/714)	(27/714)	(367/714)	(58/714)
**GA 28–29**	695	29+0/7	1180	−1.36	33.1%	−0.07	12.5%	3.2%	56.0%	1.2%
**wk**		(28+0/7 to 29+6/7)	(410–2095)	(−5.89 to 3.34)	(230/695)	(−3.09 to 2.87)	(87/695)	(22/695)	(389/695)	(8/695)
**GA 30–31**	716	30+6/7	1422	−1.67	40.4%	−0.32	17.6%	4.6%	52.9%	0.1%
**wk**		(30+0/7 to 31+6/7)	(378–2400)	(−6.62 to 4.47)	(289/716)	(−3.27 to 3.07)	(126/716)	(33/716)	(379/716)	(1/716)
**Total**	**2941**	**27+5/7**	**980**	**−1.2**	**28.5%**	**−0.11**	**12.6%**	**3.5%**	**53.8%**	**10.0%**
		(22+1/7 to 31+6/7)	(348–2400)	(−6.62–4.47)	(839/2941)	(−3.27 to 3.07)	(372/2941)	(102/2941)	(1582/2941)	(295/2941)

Abbreviations: BW, indicates birth weight; BWSDS, birth weight standard deviation score; GA, gestational age; ROP, retinopathy of prematurity; SGA, small for gestational age.; wk, weeks.

### BWSDS and GA groups

**Figure 2:**
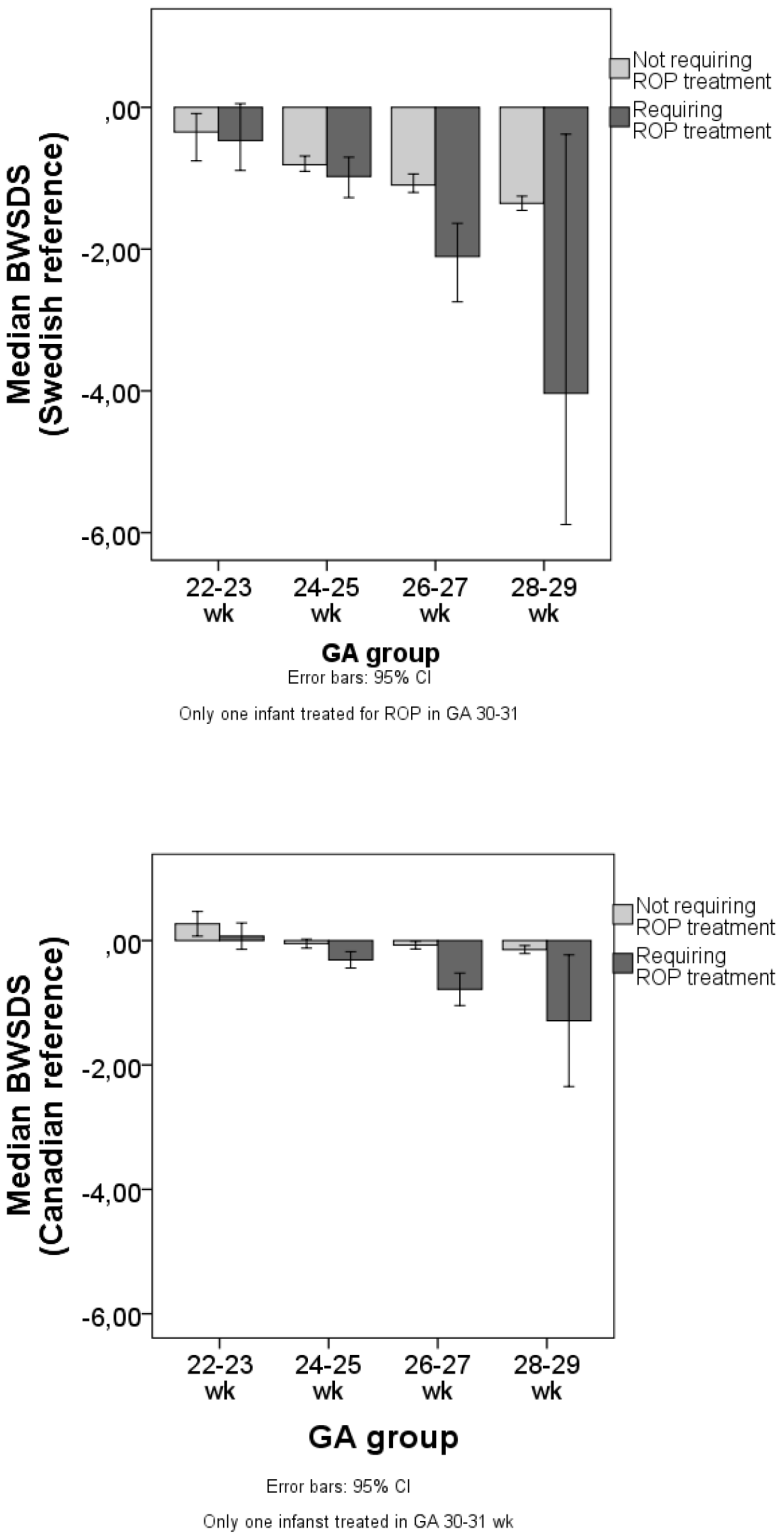
The median BWSDS of infants treated for ROP. The BWSDS was calculated according to the Swedish or Canadian growth reference within each GA group.

A negative correlation between BWSDS and GA was found regardless of growth reference; low BWSDS was associated with higher GA (Swedish reference: r_S_ = −0.25, *p*<0.001, Canadian reference r_S_ = −0.12, *p*<0.001). Median BWSDS, regardless definition, was lower in infants treated for ROP compared with infants not treated for ROP between GA 24–29 weeks (Mann-Whitney U test, *p*<0.05, Figure 2).

### Prevalence of SGA

The prevalence of SGA, regardless of definition, increased with GA. The prevalence of SGA_Sweden_ was 5.6% (7/126) in the 22–23 weeks GA group, and increased to 40.4% (289/716) in the 30–31 weeks GA group. The prevalence of SGA_Canada_ was 4.0% (5/126) in the 22–23 weeks GA group and increased to 17.6% (126/716) in the 30–31 weeks GA group (Table 1).

### Prevalence of infants treated for ROP

The prevalence of infants treated for ROP was 45.2% (57/126) in the 22–23 weeks GA group. The prevalence decreased to 0.1% (1/716) in the 30–31 weeks GA group. The prevalence of infants treated for ROP varied from 17.1% (73/426) in the EXPRESS cohort to 4.1% (14/338) in the Boston cohort (Table 1).

### Risk factors for ROP that will require treatment

Possible risk factors for an infant who will require ROP treatment were as follows: GA (weeks at birth), BW (50-g increments), gender, BWSDS and SGA calculated and defined by both the Swedish and Canadian growth references, and severe growth restriction calculated by the Canadian growth reference. First, each risk factor was evaluated separately using logistic regression. For the whole cohort, low GA at birth (*p*<0.001), low BW (*p*<0.001), and male gender (*p*<0.05) were significant risk factors. Neither low BWSDS_Sweden_ nor SGA_Sweden_ were significant risk factors for ROP that will require treatment. However, low BWSDS_Canada_ (*p*<0.001), SGA_Canada_ (*p*<0.01), and severe growth restriction (*p*<0.001) were risk factors. When dividing the infants into GA groups, BWSDS, SGA, and severe growth restriction were significant risk factors for infants born at GA 26–27 weeks independent of whether these were defined according to the Swedish or Canadian growth references (Table 2).

**Table 2:** Association between infant natal characteristics and ROP that required treatment (analyzed using univariate logistic regression analysis).

	Whole cohort	GA groups			
Clinical characteristics	GA 22–32 wk	GA 22–23 wk	GA 24–25 wk	GA 26–27 wk	GA 28–29 wk
	(*n* = 2941)	(*n* = 126)	(*n* = 690)	(*n* = 714)	(*n* = 695)
**Variable**					
**Male gender**					
**OR**	**1.30**	NS	1.50	NS	NS
**95% CI**	**1.02–1.67** *		1.05–2.13*		
**GA, wk**					
**OR**	**0.47**	NS	0.44	NS	NS
**95% CI**	**0.43–0.52** ***		0.31–0.63***		
**BW (50 g increments)**					
**OR**	**0.74**	NS	0.86	0.79	0.80
**95% CI**	**0.72–0.77** ***		0.80–0.92***	0.73–0.85***	0.69–0.93**
**BWSDS, Swedish reference**					
**OR**	**NS**	NS	0.86	0.56	0.44
**95% CI**			0.75–0.99*	0.46–0.69***	0.27–0.73**
**SGA, Swedish reference**					
**OR**	**NS**	NS	NS	3.62	NS
**95% CI**				2.10–6.25***	
**BWSDS, Canadian reference**					
**OR**	**0.78**	NS	0.70	0.40	0.24
**95% CI**	**0.68–0.89** ***		0.57–0.86**	0.29–0.54***	0.10–0.54**
**SGA, Canadian reference**					
**OR**	**1.60**	NS	1.80	4.67	12.30
**95% CI**	**1.16–2.20** **		1.08–3.01*	2.54–8.57***	2.88–52.41**
**Severe growth restriction, Canadian reference**					
**OR**	**2.43**	–	NS	7.83	21.10
**95% CI**	**1.48–3.99** ***	**–**		3.40–18.04***	4.70–94.75***

Abbreviations: BW indicates birth weight; BWSDS, birth weight standard deviation score; GA, gestational age; NS, not significant; OR, odds ratio; ROP, retinopathy of prematurity; SGA, small for gestational age; wk, weeks.

*p<0.05,

**p<0.01,

***p<0.001.

**Figure 3:**
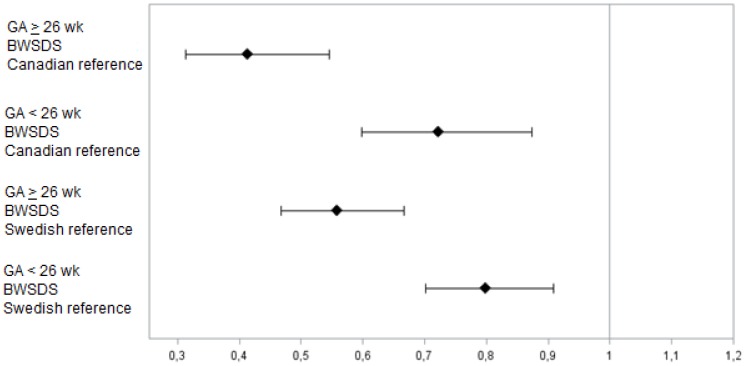
BWSDS odds ratio for infants for ROP requiring treatment in relation to immaturity. The BWSDS was calculated using the Swedish or Canadian growth chart reference for different GA cut-offs.

In the next step, multivariable logistic regression analysis was used. Low GA (*p*<0.0001), male gender (*p*<0.01 and *p*<0.05), and the interaction term (BWSDS*GA group) (*p*<0.001), regardless of BWSDS growth reference, were risk factors for ROP that will require treatment. The multivariable logistic regression analysis was performed with the Swedish as well as the Canadian growth references (Hosmer-Lemeshow test did not indicate any lack of fit for either model, p_Swe_ = 0.67, p_Can_ = 0.53). Independent of which growth reference was used, BWSDS was less important as a risk factor in infants born at GA <26 weeks compared with infants born at GA ≥26 weeks (BWSDS_Sweden_; OR = 0.80, 95% CI 0.70–0.91 *vs* OR = 0.56, 95% CI 0.47–0.68 and BWSDS_Canada_; OR = 0.72 95% CI 0.60–0.87 *vs* OR = 0.41, 95% CI 0.31–0.55) (Table 3 and Figure 3). Consequently, infants born at GA <26 weeks had reduced odds of requiring treatment by 20–28% for every 1 SD increase in BWSDS compared with infants born at GA ≥26 weeks who had a 44–59% reduction for every 1 SD increase in BWSDS.

**Table 3:** Association between infant natal characteristics and ROP that required treatment (according to multivariable logistic regression analysis).

Variable	OR	P	95% CI
BWSDS for Infants with GA <26 weeks at birth (Swedish reference)	0.80	0.0007	0.70–0.91
BWSDS for Infants with GA ≥26 weeks at birth (Swedish reference)	0.56	<0.0001	0.47–0.67
GA	0.41	<0.0001	0.35–0.48
Male gender	1.45	0.0085	1.10–1.91

OR variable	OR	*p*	95% CI
BWSDS for Infants with GA <26 weeks at birth (Canadian reference)	0.72	0.0008	0.60–0.87
BWSDS for Infants with GA ≥26 weeks at birth (Canadian reference)	0.41	<0.0001	0.31–0.55
GA	0.44	<0.0001	0.38–0.51
Male gender	1.32	0.0463	1.00–1.74

Abbreviations: BWSDS indicates birth weight standard deviation score; GA, gestational age; OR, odds ratio; ROP, retinopathy of prematurity.

## Discussion

In this study, we established that the impact of low BWSDS and SGA as risk factors for severe ROP requiring treatment is dependent on GA at birth. This result persisted even when we used two different growth charts to calculate BWSDS and define SGA. GA at birth is a strong predictor of ROP and a low BWSDS may enforce the power of this predictor in more mature infants. These findings may help to explain the inconclusive results of previous studies. The risk of developing severe ROP that requires treatment is increased for the most immature infants; growth restriction is less of a risk factor. If born at a more mature age, the preterm infant's risk is minor for severe ROP unless the infant is born growth restricted. Our new finding that low BWSDS and SGA increases the risk significantly in more mature infants will be of value when counseling parents regarding their infant's risk of developing severe ROP and planning for ROP screening. Our findings could improve the ability to identify those more mature infants at greater risk for ROP who may require treatment, and spare mature infants at minor risk at least some of the painful and stressful eye examinations.

In previous studies concerning low BWSDS as a risk factor for ROP, infants were grouped differently. This could explain why the results have been inconclusive [2], [3], [13], [14], [16]–[18]. However, Qui et al. [15] reported that the impact of SGA as a risk factor for severe ROP varies for infants born ≤26, 27–28, 29–30, or 31–32 weeks GA. Consequently, differences among study results concerning low BWSDS as a risk factor for ROP may depend on the infants' GA at birth. An additional difference in these reports is the degree of ROP investigated, which varied from “any ROP” to “severe ROP”.

Variability among the study results may also arise from the different growth charts used for calculating BWSDS, and in the definition of SGA. In the studies noted above, SGA was defined as a BW deficit of less than the 3^rd^ to below the 10^th^ percentile. In the present study, we chose to use two well-established but different growth references, both of which were developed for preterm infants from GA of 22 weeks, but constructed differently regarding the included infants. We defined SGA as less than −2 SDS of GA-appropriate BW with the Swedish reference, and SGA defined as BW below the 10^th^ percentile with the Canadian reference. We found a significantly lower median BWSDS and higher prevalence of SGA in the cohort when we used the Swedish growth reference to calculate BWSDS compared with results calculated with the Canadian growth reference. This finding is not unexpected since the Swedish reference is based on fetal ultrasound, which is considered to reflect undisturbed intrauterine growth, and the Canadian reference which is based on live and still born preterm infants, regardless cause of preterm birth or death. One must question whether strict cutoffs for prenatal growth restriction, such as SGA, are the preferred choice when estimating an infant's risk of developing severe ROP.

In univariate logistic regression for the whole cohort, BWSDS_Sweden_ and SGA_Sweden_ were not significant risk factors, whereas BWSDS_Canada_ and SGA_Canada_ were risk factors. These results show that when estimating prenatal growth restriction, the choice of the growth chart reference may affect the results. Subgroup analysis showed that SGA and BWSDS were variable risk factors dependent on GA at birth. Low BWSDS, as well as SGA, regardless of definition, had the same impact. The more mature the infant, the greater the impact of low BWSDS as a risk factor for severe ROP that will require treatment.

In multivariable logistic regression analysis, we confirmed that the odds of requiring treatment for ROP were reduced with higher BWSDS. As we designed an interaction term, the product of relevant BWSDS and GA group, we established that BWSDS (regardless of growth reference) was dependent on GA at birth. Infants born at GA <26 weeks had reduced odds of requiring treatment for ROP by 20–28% for every 1 SD increase in BWSDS compared with infants born at GA ≥26 weeks, who had a 44–59% reduction for every 1 SD increase in BWSDS (BWSDS_Sweden_; OR = 0.80 vs 0.56 and BWSDS_Canada_; OR = 0.72 vs 0.41). To our knowledge, no previous study has evaluated BWSDS in preterm infants as a risk factor for severe ROP while taking into account the interaction with GA at birth, and considering different BW deficit references.

The underlying reason for the observed shift in the impact of BWSDS as a risk factor for ROP that will require treatment should be discussed. A confounding factor of this finding may be the increasing prevalence of infants born with low BWSDS with increasing GA at birth. Infants born with low BWSDS or SGA have an increased risk for perinatal death, both fetal and neonatal [30]–[32]. Moreover, when low BWSDS and SGA is associated with additional prematurity, the perinatal mortality rates increase [33]. Stoll et al. [34] suggested that the increased survival rates with increasing GA partly reflect physicians' attitudes towards providing intensive care, as measured by the frequent use of antenatal corticosteroids as well as the frequency of active resuscitation in the delivery room [34], [35]. Consequently, more aggressive lifesaving interventions are initiated for infants born at an older GA, even for severely growth-retarded fetuses. Thus, the increasing impact of low BWSDS as a risk factor for ROP that will require treatment may be a reflection of the increasing number of surviving infants born with low BWSDS with additional GA weeks at birth. Regardless of the reason behind the finding that low BWSDS is a risk factor for ROP depending on GA, the result remains the same; infants who are born more mature but that are growth restricted should receive adequate attention from the screening ophthalmologist.

Today, most current ROP screening guidelines, which primarily use GA and BW as screening criteria, are vague about the selection of which infants should be screened. Prenatal as well as postnatal growth restriction can be identified by utilizing web-based systems such as WINROP [10], [11] and CHOP [36]. By improving the identification of infants at greatest risk for ROP that will require treatment, ophthalmological interventions can focus on those infants at greatest risk, sparing those at minor risk from at least some stressful and painful eye examinations.

In our study, male gender was a significant risk factor for ROP that will require treatment according to univariate logistic regression analysis in the whole cohort (OR = 1.30, 95% CI 1.02–1.47, *p*<0.05) and for infants born during GA 24–25 week (OR = 1.50, 95% CI 1.16–2.20, *p*<0.05). In the earliest descriptions of ROP in the 1940s, male preterm infants were described as more frequently affected by ROP than female ones [37]. A few subsequent reports have supported these findings [2], [38]. In the multivariable logistic regression analysis, male gender persisted as a risk factor. Whether or not male gender is also a risk factor for severe ROP dependent on GA will require further investigation.

A major strength of this study is that we calculated BWSDS using two different, well-established growth reference charts; one based on undisturbed intrauterine growth, and the other based on live as well as still born preterm infants. Using both reference models, low BWSDS as well as SGA were risk factors for ROP that will require treatment depending on the infants' immaturity at birth.

Another major strength is the large size of the cohort eligible for assessment, which included 2941 infants from three countries; Canada, Sweden, and the USA. With this constellation of study cohorts, we successfully enrolled almost 700 infants in each pairwise GA group, with the exception of the most preterm group, GA 22–23 weeks.

A limitation of this study is that other established risk factors were not considered when calculating the risk of ROP that will require treatment such as genetic disorders, twin situation, race, and other postnatal morbidities such as days of ventilation, bronchopulmonary dysplasia, septicemia, and necrotizing enterocolitis. Further studies of BWSDS and other postnatal morbidities as risk factors for severe ROP, with special attention to correlations with the infants' GA at birth, would be of great interest.

In summary, growth restriction at birth, calculated using two differently defined well-established growth references, is an important risk factor for ROP that will require treatment, and was dependent on the infants' degree of immaturity. ROP screening criteria need to be continually revised according to new findings in order to focus attention on those infants at greatest risk for severe ROP, and to spare infants at reduced risk from at least some of the stressful eye screening examinations.
